# Molecular characterization of extensively drug-resistant hypervirulent *Pseudomonas aeruginosa* isolates in China

**DOI:** 10.1186/s12941-024-00674-7

**Published:** 2024-02-12

**Authors:** Jun Li, Mengli Tang, Zhaojun Liu, Yuhan Wei, Fengjun Xia, Yubing Xia, Yongmei Hu, Haichen Wang, Mingxiang Zou

**Affiliations:** 1 Department of Clinical Laboratory, Xiangya Hospital, Central South University, Changsha, Hunan 410008 China; 2 National Clinical Research Center for Geriatric Disorders, Xiangya Hospital, Central South University, Changsha, Hunan 410008 China

**Keywords:** *Pseudomonas aeruginosa*, Extensive drug resistance, Clinical features, Virulence, Drug resistance mechanism, Molecular epidemiology

## Abstract

**Background:**

Recently, extensively drug-resistant *Pseudomonas aeruginosa* (XDR-PA) isolates have been increasingly detected and posed great challenges to clinical anti-infection treatments. However, little is known about extensively resistant hypervirulent *P. aeruginosa* (XDR-hvPA). In this study, we investigate its epidemiological characteristics and provide important basis for preventing its dissemination.

**Methods:**

Clinical XDR-PA isolates were collected from January 2018 to January 2023 and identified using matrix-assisted laser desorption/ionization–time-of-flight mass spectrometry; antibiotic susceptibility testing was performed by broth microdilution method, and minimum inhibitory concentrations (MICs) were evaluated. Virulence was evaluated using the *Galleria mellonella* infection model; molecular characteristics, including resistance genes, virulence genes, and homology, were determined using whole-genome sequencing.

**Results:**

A total of 77 XDR-PA strains were collected; 47/77 strains were XDR-hvPA. Patients aged > 60 years showed a significantly higher detection rate of XDR-hvPA than of XDR-non-hvPA. Among the 47 XDR-hvPA strains, 24 strains carried a carbapenemase gene, including *bla*_GES−1_ (10/47), *bla*_VIM−2_ (6/47), *bla*_GES−14_ (4/47), *bla*_IMP−45_ (2/47), *bla*_KPC−2_ (1/47), and *bla*_NDM−14_ (1/47). *ExoU*, *exoT*, *exoY*, and *exoS*, important virulence factors of PA, were found in 31/47, 47/47, 46/47, and 29/47 strains, respectively. Notably, two XDR-hvPA simultaneously co-carried *exoU* and *exoS*. Six serotypes (O1, O4–O7, and O11) were detected; O11 (19/47), O7 (13/47), and O4 (9/47) were the most prevalent. In 2018–2020, O4 and O7 were the most prevalent serotypes; 2021 onward, O11 (16/26) was the most prevalent serotype. Fourteen types of ST were detected, mainly ST235 (14/47), ST1158 (13/47), and ST1800 (7/47). Five global epidemic ST235 XDR-hvPA carried *bla*_GES_ and showed the MIC value of ceftazidime/avibactam reached the susceptibility breakpoint (8/4 mg/L).

**Conclusions:**

The clinical detection rate of XDR-hvPA is unexpectedly high, particularly in patients aged > 60 years, who are seemingly more susceptible to contracting this infection. Clonal transmission of XDR-hvPA carrying *bla*_GES_, which belongs to the global epidemic ST235, was noted. Therefore, the monitoring of XDR-hvPA should be strengthened, particularly for elderly hospitalized patients, to prevent its spread.

**Supplementary Information:**

The online version contains supplementary material available at 10.1186/s12941-024-00674-7.

## Background

*Pseudomonas aeruginosa* (PA) is a leading cause of healthcare-associated infections, including pneumonia, intra-abdominal infection, urinary tract infection, surgical site infection, and bloodstream infections [1, 2]. The drug resistance rate of *P. aeruginosa* has recently increased because of the global spread of extensively drug-resistant (XDR) and multidrug-resistant (MDR) isolates, which are associated with treatment failure and increased mortality [3–5]. In China, the resistance rate of *P. aeruginosa* to carbapenems was ∼30%, and the related 30-day crude mortality was ∼40.0% [5, 6]. Therefore, in 2017, carbapenem-resistant *P. aeruginosa* was classified as a “priority one” pathogen for new antibiotics by the World Health Organization (WHO) [7]. Intrinsic resistance, chromosomal gene mutations, and transferable resistance determinants are responsible for this increasing threat, such as carbapenemases (GES, KPC, VIM, and IMP enzymes) and co-transferred aminoglycoside-modifying enzyme determinants [e.g., AAC (3′), AAC (6′), and ANT (2′)-I] [4].

Notably, recent clinical studies have reported the emergence of hypervirulent *P. aeruginosa* (hvPA). Zhang et al. reported the emergence and recurrence of KPC-producing hvPA ST697 and ST463 between 2010 and 2021 in China [8]. In vivo acquisition of *bla*_KPC-2_ in *bla*_AFM-1_-expressing hvPA ST463 was also reported in a patient with hematologic malignancy [9]. Early detection of a KPC-2-producing hvPA ST235 was reported by de Paula-Petroli et al. in Brazil [10]. These studies suggest that the reports on hvPA currently are mainly sporadic case reports, and relevant research on extensively drug-resistant *P. aeruginosa* (XDR-PA) is still lacking. Therefore, this study intended to (i) collect and screen extensively drug-resistant hypervirulent *P. aeruginosa* (XDR-hvPA) from clinical isolates, (ii) analyze the possible risk factors leading to its infection, and (iii) investigate its molecular epidemiological characteristics.

## Methods

### Bacterial strains and species identification

All non-repetitive clinically isolated XDR-PA were collected from January 2018 to January 2023 at Xiangya Hospital of Central South University, China; this is a large hospital with 3500 beds and more than 3 million outpatients every year. Matrix-assisted laser desorption/ionization–time-of-flight mass spectrometry (MALDI–TOF MS; Bruker Daltonics GmbH, Bremen, Germany) was used to identify all isolates, with *Escherichia coli* ATCC 25922 (National Center for Clinical Laboratories, Beijing, China) as the quality control strain.

### Antimicrobial susceptibility testing

The classic broth microdilution test was used to determine the minimum inhibitory concentrations (MICs) of antimicrobial agents (Hangzhou Kangtai Biotechnology Co.), including piperacillin (PRL), ceftazidime (CAZ), cefepime (FEP), aztreonam (ATM), meropenem (MEM), imipenem (IPM), amikacin (AK), gentamicin (GEN), tobramycin (TOB), ciprofloxacin (CIP), levofloxacin (LEV), piperacillin/tazobactam (TZP), nitrofurantoin (F), ceftazidime/avibactam (CZA), and polymyxin B (PB). *P. aeruginosa* ATCC 27853 (National Center for Clinical Laboratories, Beijing, China) was considered the quality control strain. The susceptibility breakpoints were interpreted using the guidelines of the Clinical and Laboratory Standards Institute (2022) [11].

The extensively drug-resistant (XDR) isolate was defined to be non-susceptible to ≥ 1 agent in all but ≤ 2 categories (i.e., bacterial isolates remain susceptible to only one or two categories) [12].

### Galleria mellonella infection model

The virulence of all collected clinical isolates was evaluated using the *G. mellonella* infection model (Tianjin Huiyude Biotech Company, Tianjin, China) [13]. In brief, 10 µL of *P. aeruginosa* overnight cultures adjusted to 1 × 10^6^ CFU/mL in physiological saline were injected into *G. mellonella* larvae, followed by 5 days of incubation in the dark at 37 °C. The survival rate was measured using PBS as the negative group, and all experiments were done in triplicate [13].

### Whole-genome sequencing and analysis

Genomic DNA was extracted from XDR-hvPA strains using TIANamp Bacteria DNA Kit (Tiangen Biochemical Technology Co., Ltd, Beijing) for NovaSeq 6000 sequencing. After passing through fastp filters and FastQC, clean reads were assembled and corrected using Unicycler to obtain the final genome sequence. Sequence typing (ST) and O serotype of XDR-hvPA strains were confirmed by multilocus sequence typing and PAst 1.0 (https://cge.cbs.dtu.dk/services/PAst/), respectively. To detect and characterize antimicrobial-resistant genes, the basic local alignment search tool (BLAST) alignments were conducted using the comprehensive antibiotic resistance database (https://card.mcmaster.ca/). All sequenced genomes were aligned to PAO1 (GenBank accession: NC_002516.2) to determine single-nucleotide polymorphisms (SNPs). Additionally, core genomes, single-nucleotide polymorphisms (cg-SNPs), and the phylogenetic tree were analyzed using Snippy (https://github.com/tseemann/snippy) and visualized using iTOL (https://itol.embl.de/) and ChiPlot (https://www.chiplot.online/). Notably, the sequence data have been deposited in NCBI with the accession number PRJNA1018421.

### Statistical analyses

SPSS 26.0 was used for statistical analysis. The underlying clinical characteristics, underlying diseases, invasive procedure (e.g., urinary catheter, gastric tube, and peripherally inserted central catheter), and previous antibiotic exposure of XDR-hvPA and XDR-non-hvPA strains were compared. The Fisher’s exact test or *χ*^*2*^ test was used for categorical variables, and the Student’s *t*-test was used for continuous variables. A *P* value of < 0.05 indicated statistical significance.

## Results

### Collection of XDR-PA strains and screening of XDR-hvPA

A total of 77 non-repetitive *P. aeruginosa* strains were isolated from various types of clinical specimens taken from 77 patients; five of these patients were emergency patients, and the remainder were hospitalized patients. In terms of the distribution of patients in departments, most of the patients were in the intensive care unit (ICU, 40.3%, 31/77), followed by integrated Chinese and Western medicine departments (ICWM, 22.0%, 17/77), rehabilitation departments [11.7% (9/77)], emergency departments [6.5% (5/77)], respiratory departments [3.9% (3/77)], and other departments [15.6% (12/77)]. Among the sample sources, 85.7% (66/77) were respiratory specimens, followed by urine specimens [5.2% (4/77)], fecal specimens [3.9% (3/77)], wound secretion specimens [3.9% (3/77)], and tissue specimen [1.3% (1/77)].

According to the results of antimicrobial susceptibility tests, all 77 strains were susceptible only to polymyxin B, indicating XDR-PA. Notably, the drug sensitivity results of 14 ST235 strains carrying the *bla*_GES_ gene to CZA showed that the MIC value was 2/4∼8/4 mg/L, and five strains (P54, P62, P68, P71, and P88) showed the MIC value of CZA at 8/4 mg/L, which was close to the resistance cutoff point (MIC break point for sensitivity: ≤8/4 mg/L).

**Fig. 1:**
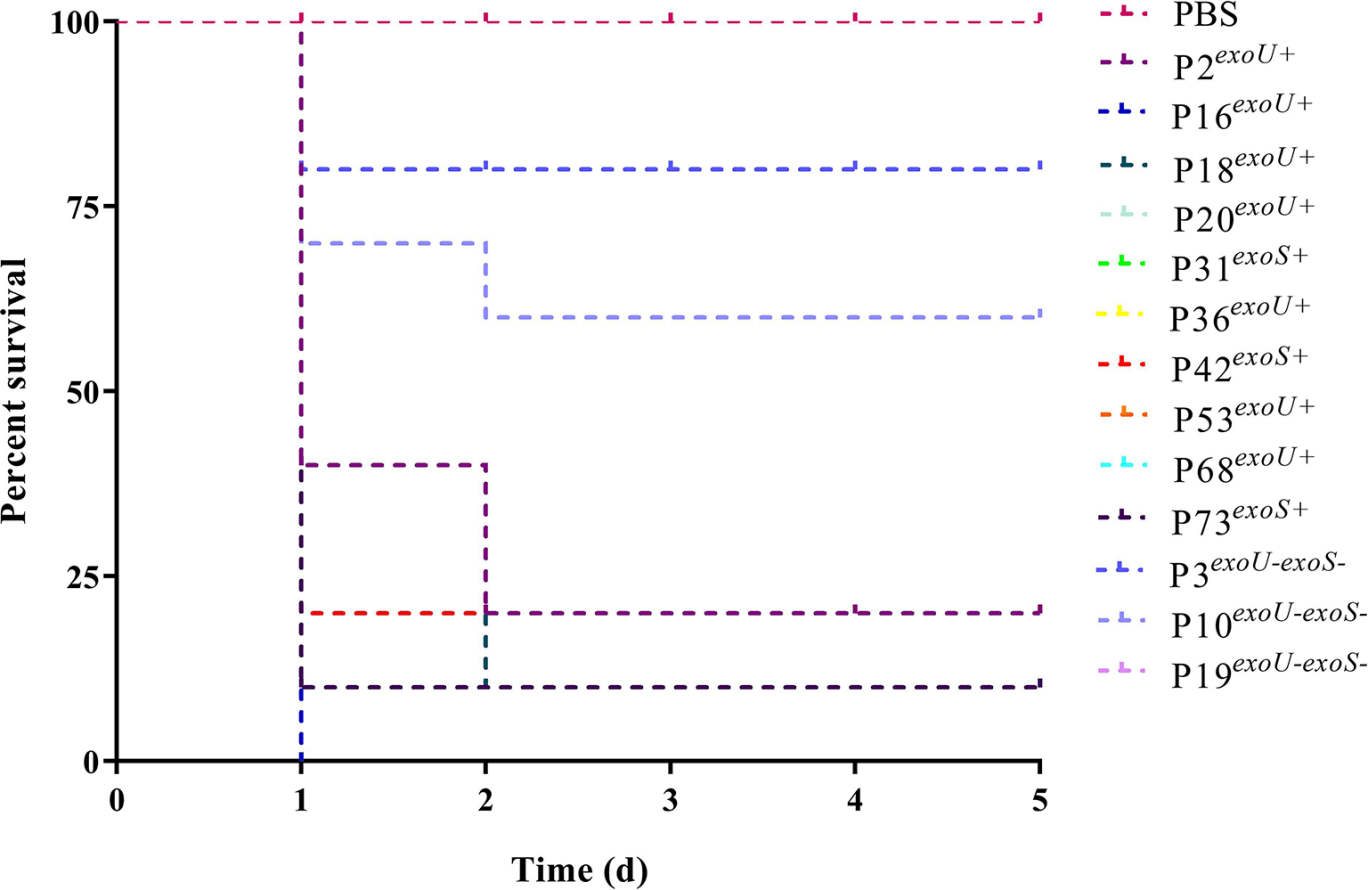
The virulence of 77 XDR-PA isolates. The virulence characterization in the *G. mellonella* infection model. PBS was used as the negative group. After infection with XDR-PA isolates carrying *exoE*/*exoS* virulence-related genes, the mortality rate of the *G. mellonella* was significantly higher

In our study, the *G. mellonella* infection model and the important virulence-related genes (*exoU* and *exoS*) were used to evaluate the virulence of all collected strains, and the results suggested that 47 strains were XDR-hvPA. (Fig. 1).

### Clinical characterizations of XDR-hvPA and XDR-non-hvPA

To clarify the clinical characteristics of XDR-hvPA and XDR-non-hvPA, a comparative analysis was conducted on these indicators, including demographic information, ICU admission, underlying diseases, invasive procedures, and patients’ history of antibiotic exposure. Patients were divided into three age groups: ≤18 years, 18–59 years, and ≥ 60 years. In patients aged ≥ 60 years, the detection rate of XDR-hvPA was significantly higher than that of XDR-non-hvPA (72.3% vs. 40.0%, *P* = 0.005); however, the rate did not differ significantly in the other two age groups. There was no significant difference in sex, length of hospitalization < 30 days, ICU admission, underlying diseases, invasive procedures, and previous antibiotic exposure. It was suggested that elderly patients were more likely to contract XDR-hvPA infection (Table 1).

**Table 1:** Clinical characteristics of infection patients caused by XDR-hvPA and XDR-non-hvPA

Variables	XDR-hvPA (*n* = 47, 61.0%)	XDR-non-hvPA (*n* = 30, 39.0%)	*P* value
Age (years)			
≤ 18	2 (4.3%)	5 (16.7%)	0.150
19–59	11 (23.4%)	13 (43.3%)	0.066
≥ 60	34 (72.3%)	12 (40.0%)	**0.005**
Male	35 (74.5%)	26 (86.7%)	0.198
Hospital stay (< 30 days)	29 (61.7%)	23 (76.7%)	0.171
ICU^a^ admission	19 (40.4%)	12 (40.0%)	0.970
**Underlying diseases**			
Chronic lung disease	23 (48.9%)	19 (63.3%)	0.216
Diabetes mellitus	9 (19.1%)	4 (13.3%)	0.506
Hypertension	17 (36.2%)	9 (30.0%)	0.577
Hyperlipidemia	4 (8.5%)	0 (0)	0.265
**Invasive procedure**			
Gastric tube	42 (89.4%)	25 (83.3%)	0.675
PICC^b^	33 (70.2%)	15 (50.0%)	0.074
Urinary catheter	31 (66.0%)	19 (63.3%)	0.814
Other	19 (40.4%)	10 (33.3%)	0.531
**Previous antibiotic exposure**			
Carbapenems	14 (29.8%)	11 (36.7%)	0.530
Quinolones	9 (19.1%)	10 (33.3%)	0.159
Aminoglycosides	8 (17.0%)	2 (6.7%)	0.332
β-enzyme inhibitor	28 (59.6%)	16 (53.3%)	0.589
Polymyxin B	4 (8.5%)	2 (6.7%)	1.000
Cephalosporin	6 (12.8%)	5 (16.7%)	0.886

^a^ICU: intensive care unit

^b^PICC: peripherally inserted central catheter

### Resistance and virulence genes of XDR-hvPA strains

**Fig. 2:**
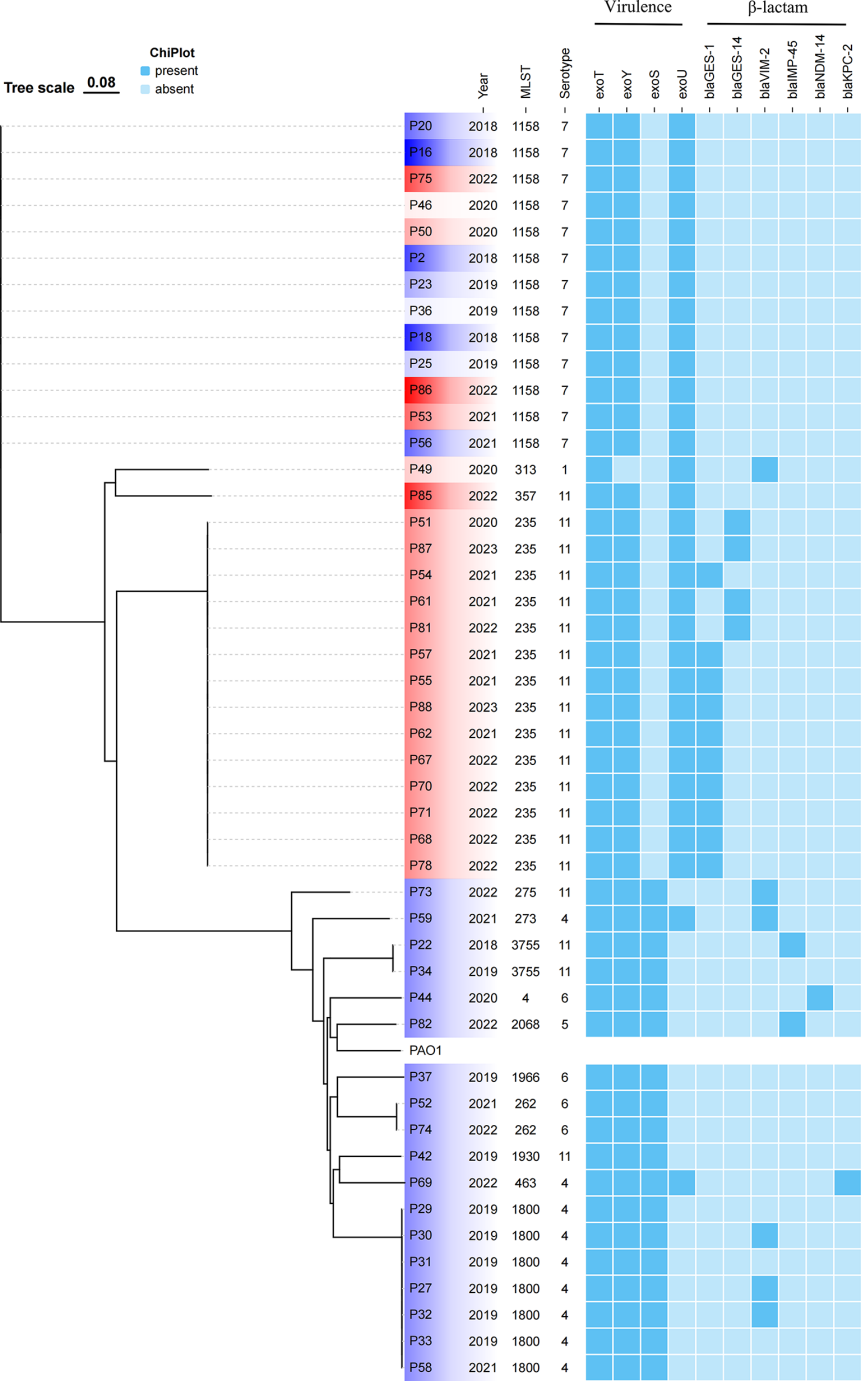
Molecular characteristics of 47 XDR-hvPA strains. PAO1 (GenBank accession: NC_002516.2) was used as the reference strain

Among 47 XDR-hvPA strains, 51.1% (24/47) strains carried the carbapenemase gene, suggesting that carbapenem-producing played a major role in drug resistance of *P. aeruginosa*. Six types of carbapenem-resistant genes were detected, namely *bla*_GES-1_, *bla*_VIM-2_, *bla*_GES-14_, *bla*_IMP-45_, *bla*_KPC-2_, and *bla*_NDM-14_, with positivity rates of 21.3% (10/47), 12.8% (6/47), 8.5% (4/47), 4.3% (2/47), 2.1% (1/47), and 2.1% (1/47), respectively. Notably, some resistance genes are rare types of genes in XDR-hvPA, such as *bla*_IMP-45_, *bla*_KPC-2_, and *bla*_NDM-14_. (Fig. 2). In addition, these strains also co-carried other drug resistance-related genes, as shown in Figure S1.

The detection rates of the virulence-associated genes *exoT*, *exoY*, *exoU*, and *exoS*, which are the important effectors of the type III secretion system of *P. aeruginosa*, were 100.0% (47/47), 97.9% (46/47), 66.0% (31/47), and 38.3% (18/47), respectively. Interestingly, two strains (P59 and P69) co-carried *exoU* and *exoS* simultaneously (Fig. 2). Genes encoding flagella, type IV pili and non-pilus adhesins, and extracellular virulence factors were also detected (Figure S2).

### Homology analysis of XDR-hvPA

Notably, 47 XDR-hvPA strains belonged to 14 ST types, with ST235 (*n* = 14, 29.8%) being the most prevalent, followed by ST1158 (*n* = 13, 27.7%) and ST1800 (*n* = 7, 14.9%). All strains of ST235 carried the *bla*_GES_ gene; ST1158 did not carry the carbapenemase gene, and three strains of ST1800 carried the *bla*_VIM-2_ gene. The strain carrying the *bla*_NDM-14_ gene belonged to ST4; the strain carrying *bla*_IMP-45_ belonged to ST2068 and ST3755, and the strain carrying the *bla*_KPC-2_ gene belonged to ST463. The 47 XDR-hvPA strains were divided into 17 clusters. Interestingly, the isolate carrying the *bla*_GES_ gene (ST235) was identified to belong to the same cluster with the difference in SNP from 2 to 51 (mainly from 2021 to 2022), indicating the existence of clonal transmission (Fig. 2).

**Fig. 3:**
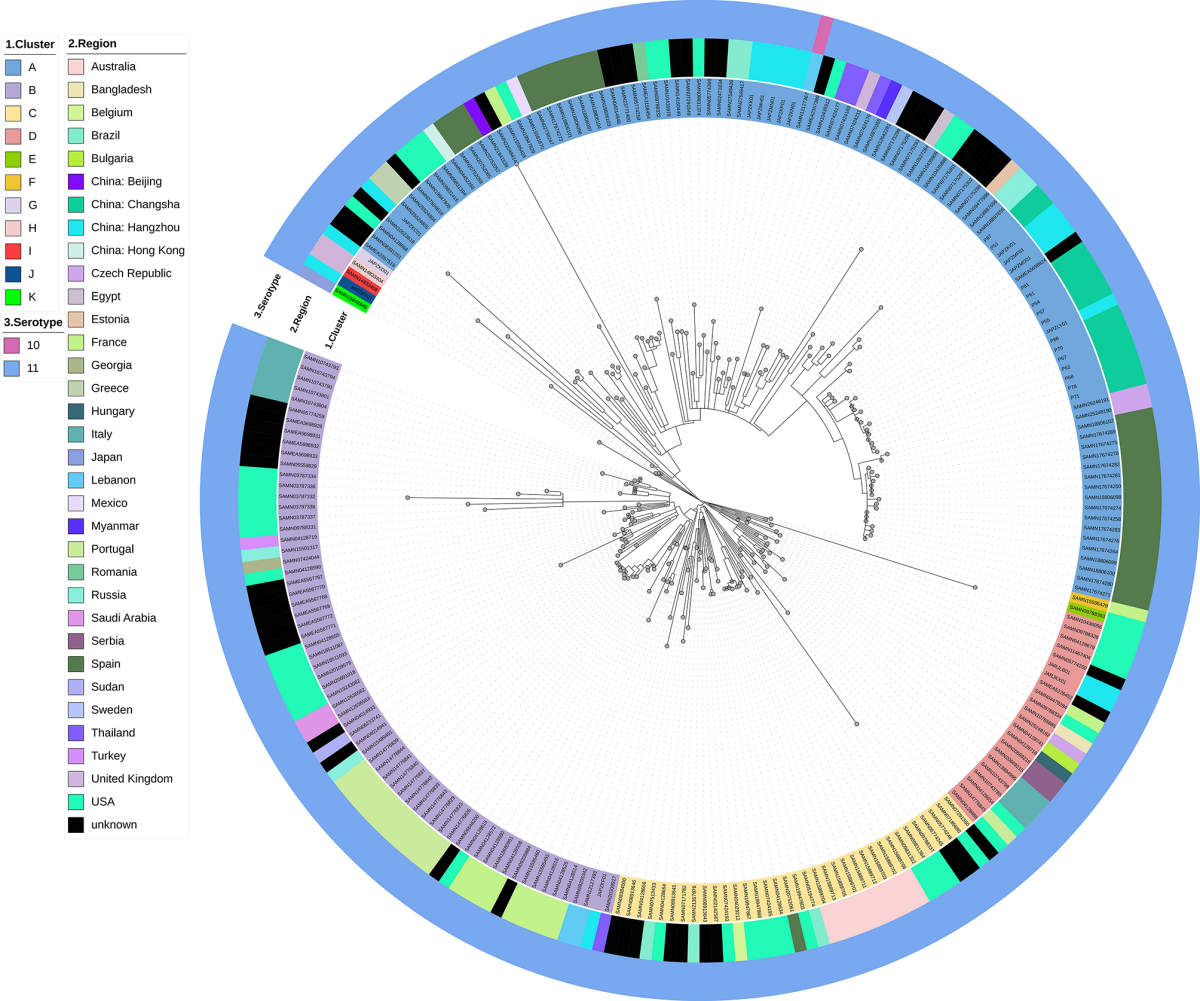
The phylogenetic analyses of ST235 PA from the global region. The first circle represents the cluster; the second circle shows the countries or regions where the strains were distributed; the third circle indicates the O serotype. P54 in our study was used as the reference strain

We downloaded the genetic data of global epidemic ST235 *P. aeruginosa* from the *Pseudomonas* genome database (https://www.pseudomonas.com/) and analyzed the evolutionary relationship between ST235 *P. aeruginosa* globally and XDR-hvPA strains in this study. Thus far, 206 strains of ST235 *P. aeruginosa* have been uploaded to the system globally. In addition, we conducted homology analysis on these 206 strains of ST235 *P. aeruginosa*, 15 strains of ST235 *P. aeruginosa* from Hangzhou, Zhejiang, which were previously reported by Li et al. [14], and 14 strains of XDR-hvPA reported in this study. The results showed that these 235 strains could be divided into 11 clusters, with clusters A, B, C, and D being the most prevalent, accounting for 98.3% of all strains, indicating clonal transmission. Fourteen XDR-hvPA strains were classified into A clone cluster. These had the closest similarity to JAPZLY01, which were obtained from Hangzhou, Zhejiang, in a previous report by Li et al. [14], with an SNP difference of 1–5 (except for P51 and P87), indicating that ST235 *P. aeruginosa* may have clonal transmission in China and should be highly valued. (Fig. 3).

### Evolution relationship of serotype in the past six years

This study analyzed the change of O serotype during the evolution of the strain. A total of six O serotypes of 47 XDR-hvPA strains were detected, namely O1, O4, O5, O6, O7, and O11. Among them, O11 (40.4%, 19/47), O7 (27.6%, 13/47), and 04 (19.1%, 9/47) accounted for the highest proportion. From 2018 to 2020, O4 and O7 were the prevalent serotypes, whereas in the last three years, O11 was the most prevalent serotype, accounting for 61.5% of all serotypes (16/26). (Fig. 2). Notably, 235 strains of ST235 *P. aeruginosa* isolated globally were O11, except one strain (SAMN12127367) was O10. (Fig. 3).

## Discussion

*P. aeruginosa* has long been recognized as a significant opportunistic pathogen for hospital infections, and in recent years, the number of resistant isolates, particularly MDR and XDR isolates, has increased. These isolates can cause treatment failure and higher mortality [3–5]. Moreover, hvPA has been an emergency in clinical in recent years [8–10]. However, it is currently unclear whether this hypervirulent strain also exists in XDR-PA. This study aimed to investigate the clinical and molecular characteristics of XDR-hvPA to establish a laboratory foundation for the efficient management of these isolates.

Owing to its ease of operation and preservation benefits, the *G. mellonella* infection model has been extensively used to evaluate bacterial virulence [15–18]. In this study, the virulence of all collected strains was evaluated using this model and the important virulence-related genes (*exoU* and *exoS*), and 61.0% of strains were found to be XDR-hvPA, suggesting that the detection rate of highly virulent strains in XDR-PA was much higher than expected. In addition, patients aged ≥ 60 years old were more vulnerable to XDR-hvPA infection, and this age is also considered an independent risk factor for healthcare-associated infections (HAIs) [19]. Therefore, such infections in elderly patients require more attention. Previous studies suggested that prior ICU hospitalization, history of invasive operation(s), and previous antibiotic exposure (e.g., carbapenems and cephalosporin) may be independent risk factors for *P. aeruginosa* infection [5, 20, 21]. However, no significant differences were noted between XDR-hvPA and XDR-non-hvPA in this study, which may be related to the serious condition of these patients, the use of multiple antibiotics, and a history of invasive procedures.

Carbapenem-resistant *P. aeruginosa* usually shows resistance to the vast majority of clinical antibiotics and even XDR due to the production of carbapenemase, high efflux pump expression, and deletion of outer membrane protein. In our study, most XDR-hvPA strains were carbapenemase producers, suggesting that the production of this enzyme is the main mechanism leading to their drug resistance, which is inconsistent with previous research [22–24]; this inconsistency can be attributed to all strains in this study being XDR and plasmids carrying multidrug resistance genes being obtained from outside. It is worth noting that among these carbapenemases in XDR-PA in our study, *bla*_GES_ was the most prevalent, which is mainly detected in *P. aeruginosa* [25, 26]. Recent research has shown that increased *bla*_GES-1_ expression caused by the class 1 integron’s potent promoter reduces CRPA susceptibility to ceftazidime–avibactam [14, 27]. Although all strains carrying *bla*_GES-1_ in this study were not resistant to CZA, the MIC value of CZA in five strains had reached the susceptibility breakpoint (8/4 mg/L). This study also found that in addition to *bla*_VIM-2_ commonly seen in *P. aeruginosa*, some rare carbapenemase genotypes, such as *bla*_KPC-2_ and *bla*_IMP-45_, were also detected. The *bla*_KPC_ first detected in *Klebsiella pneumoniae* has been worldwide dissemination in this strain [28, 29]. In recent years, it has come to be increasingly found in *P. aeruginosa*, and some reports have shown that the KPC gene is also found in hvPA, which was mainly in the southeastern coastal areas of China and predominantly in a potentially high-risk clone of *P. aeruginosa* ST463 [8, 9, 30, 31]. De Paula-Petroli et al. also reported a KPC-2-producing hvPA ST235 in Brazil [10]. This study is the first to detect hvPA ST463 carrying KPC in the central southern region, indicating that it may have spread from coastal areas to the central region, which is a noteworthy finding. In addition, a previously rare drug resistance gene, *bla*_NDM-14_, was also detected in XDR-hvPA. Certainly, these strains also carried multiple other drug resistance-related genes, as shown in Figure S1.

The virulence of *P. aeruginosa* may be related to its many flagella, type IV fimbriae, and non-fimbriae adhesins [32]. *ExoU*, *exoT*, *exoY*, and *exoS*, the four key effectors in the type III secretion system, can help inject toxic proteins into host cells and are also closely related to the virulence of *P. aeruginosa* [18, 30]. In our study, only one strain did not carry *exoY*, whereas all others carried *exoT* and *exoY.* Previous studies have shown mutual exclusion of the *exoU* and *exoS* genes [33, 34], and *exoU*-positive strains presented multiple resistance mechanisms and stronger virulence in *G. mellonella* [13]. The *exoU* and/or *exoS* genes were positive in XDR-hvPA, and two strains co-carried *exoU* and *exoS* genes simultaneously, which may increase drug resistance and virulence [35].

Although *P. aeruginosa* has a nonclonal epidemic nature, some genotypes are found to be linked to global outbreaks, including ST111, ST175, ST235, ST244, and ST395 [36]. ST235, which is the most common of these high-risk clones, has been linked to poor clinical outcomes, partly due to high levels of antibiotic resistance [4, 36], which was also detected in our XDR-hvPA carrying *bla*_GES_ with clonal distribution. In addition, phylogenetic analyses of global ST235 *P. aeruginosa* were performed, and the results showed that ST235 *P. aeruginosa* was mainly divided into four large clusters and had clonal transmission. The 14 XDR-hvPA strains in this study were highly correlated with JAPZLY01 from the report in Zhejiang Province by Li et al. [14], with minimal SNP differences (ranging from 1 to 5), suggesting that ST235 *P. aeruginosa* also has clonal transmission in China and deserves clinical attention. This study also identified the existence of clone propagation in ST1158 and ST1800 XDR-hvPA.

Our study found six O serotypes in XDR-hvPA strains, and O11, O7, and O4 accounted for the highest proportion. Notably, O4 and O7 were the prevalent serotypes from 2018 to 2020, whereas O11 was the prevalent serotype in recent years, accounting for 61.5% of all serotypes (16/26), indicating that the main serotype may have changed from O4 and O7 to O11. It is worth noting that 235 strains of ST235 *P. aeruginosa* isolated globally are O11, except one strain (SAMN12127367), which is O10. This suggests that the O antigen serotype may be closely related to high-risk clones and virulence. According to Del Barrio-Tofio et al., there is a close connection among the *P. aeruginosa* O antigen serotypes, resistance profiles, and high-risk clones. For example, O4 is associated with ST175’s MDR/XDR profile [37]. However, we found that O4 mainly belonged to ST1800 XDR-hvPA and a *bla*_KPC-2_-carried ST463 isolate, which were also found in other regions in China [8, 9, 30, 31].

## Conclusions

In summary, the clinical detection rate of XDR-hvPA may have exceeded our expectations, particularly for hospitalized patients aged over 60 years, and this age may be an important risk factor for increased vulnerability to this infection. There is a clonal transmission of XDR-hvPA carrying the GES-type carbapenemase, which belongs to the global epidemic ST235. To effectively prevent such transmission, it is necessary to strengthen the monitoring of XDR-hvPA in hospitalized patients, particularly the elderly.

### Electronic supplementary material

Below is the link to the electronic supplementary material.

Supplementary Material 1: Figure S1. Heatmap of XDR-hvPA carrying resistance genes; Figure S2. Heatmap of XDR-hvPA carrying virulence genes

## Data Availability

The datasets generated and analysed during the current study are available from the corresponding author on reasonable request.

## Abbreviations

PA: *Pseudomonas aeruginosa*
XDR: extensively drug-resistant
MDR: multidrug-resistant
WHO: World Health Organization
hvPA: hypervirulent *P. aeruginosa*
XDR-PA: extensively drug-resistant *P. aeruginosa*
XDR-hvPA: extensively drug-resistant hypervirulent *P. aeruginosa*
MALDI–TOF MS: matrix-assisted laser desorption/ionization–time-of-flight mass spectrometry
MICs: minimum inhibitory concentrations
ST: sequence typing
PRL: piperacillin
CAZ: ceftazidime
FEP: cefepime
ATM: aztreonam
MEM: meropenem
IPM: imipenem
AK: amikacin
GEN: gentamicin
TOB: tobramycin
CIP: ciprofloxacin
LEV: levofloxacin
TZP: piperacillin/tazobactam
F: nitrofurantoin
CZA: ceftazidime/avibactam
PB: polymyxin B
BLAST: basic local alignment search tool
SNPs: single-nucleotide polymorphisms
ICU: intensive care unit
PICC: peripherally inserted central catheter
HAIs: healthcare-associated infections
