# Supplementary material for: Molecular characterization of extensively drug-resistant hypervirulent Pseudomonas aeruginosa isolates in China
Source: Ann Clin Microbiol Antimicrob. 2024 Feb 12;23:13. doi: 10.1186/s12941-024-00674-7 (PMC10863134; doi:10.1186/s12941-024-00674-7)
Supplement: Supplementary file 1 — Supplementary Material 1: Figure S1. Heatmap of XDR-hvPA carrying resistance genes; Figure S2. Heatmap of XDR-hvPA carrying virulence genes [file 12941_2024_674_MOESM1_ESM.docx]

**Supplementary Materials**


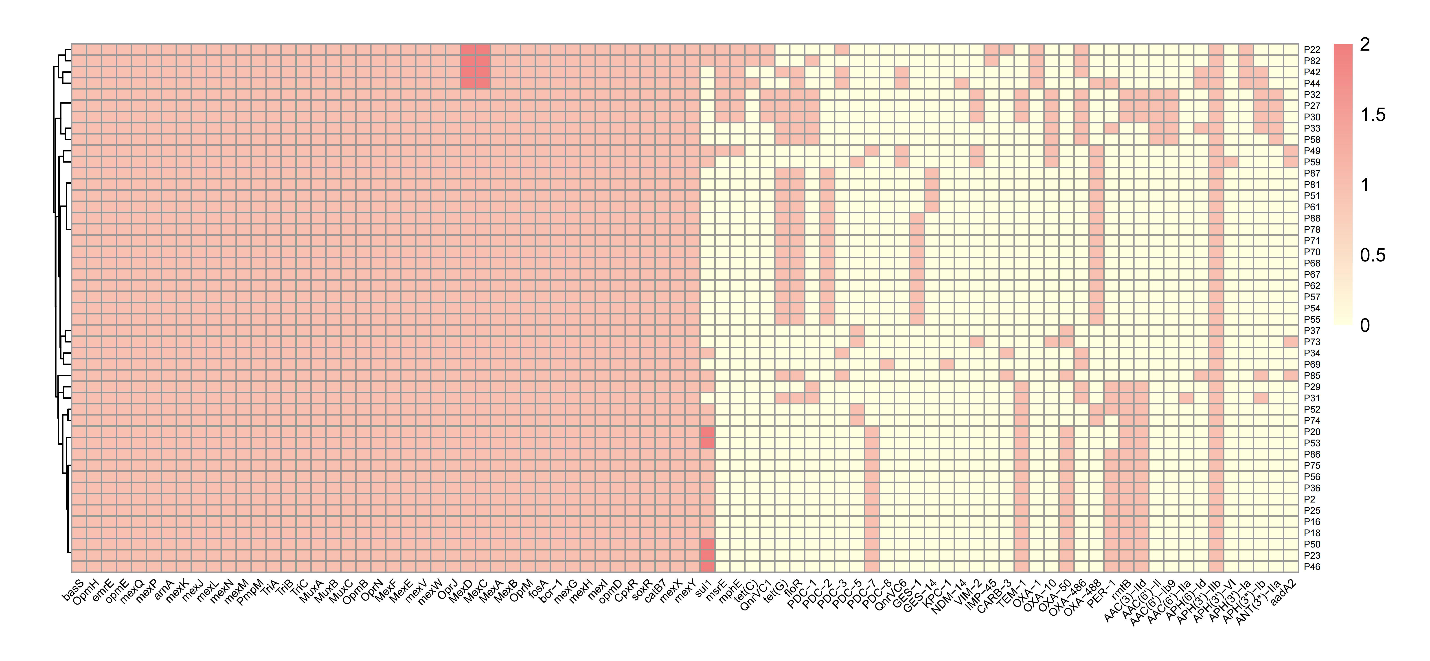


**Figure S1. Heatmap of XDR-hvPA carrying resistance genes**

Red indicates carrying the gene, and light yellow indicates not carrying the gene.


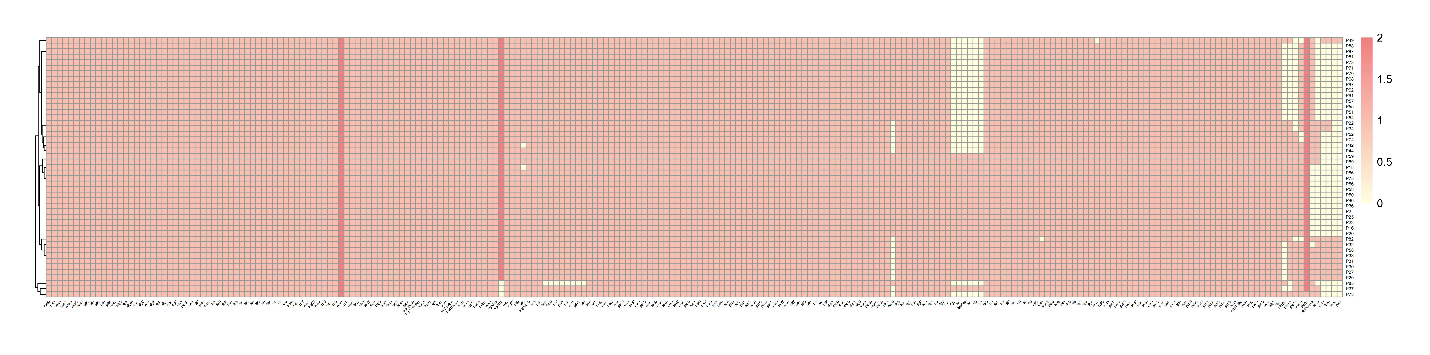


**Figure S2. Heatmap of XDR-hvPA carrying virulence genes**

Red indicates carrying the gene, and light yellow indicates not carrying the gene.
